# Rutin attenuates Sorafenib-induced Chemoresistance and Autophagy in Hepatocellular Carcinoma by regulating BANCR/miRNA-590-5P/OLR1 Axis

**DOI:** 10.7150/ijbs.62471

**Published:** 2021-08-19

**Authors:** Meng Zhou, Gan Zhang, Jun Hu, Yanzhi Zhu, Haoming Lan, Xianfeng Shen, Yi Lv, Linsheng Huang

**Affiliations:** 1 Department of Hepatopancreatobiliary Surgery, Taihe Hospital, Hubei University of Medicine, Shiyan, Hubei 442000, P. R. China.; 2 Department of Hepatobiliary Surgery, The First Affiliated Hospital, Xi'an Jiaotong University, Xi'an, Shaanxi 710000, P. R. China.

**Keywords:** Rutin, Hepatocellular carcinoma, Drug resistance, Autophagy, Non-coding RNA

## Abstract

Rutin, the main component of Potentilla discolor Bunge, was proven to exhibit anti-tumor properties. Sorafenib (SO) is conventionally used in chemotherapy against hepatocellular carcinoma (HCC), but acquired resistance developed during long-term therapy limits its benefits. This study aimed to explore the molecular mechanism of rutin in SO-induced autophagy and chemoresistance in HCC. Sixty-eight paired HCC patients who received the same chemotherapy treatment were obtained. We also established two SO resistance cell lines and then utilized high-throughput RNA sequencing to explore their long non-coding RNA (lncRNA) expression profiles. The target microRNA (miRNA) and downstream mRNA were also explored. Our results indicated that rutin treatment attenuates autophagy and BANCR expression in SO resistance cells. Transmission electron microscopy clearly showed a significantly decreased number of autophagosomes after rutin-treated HepG2/SO and HCCLM3/SO cells. BANCR knockdown promotes the sensitivity of SO resistance cells to SO. Further study found that BANCR acts as a molecular sponge of miR-590-5P to sequester miR-590-5P away from oxidized low-density lipoprotein receptor 1 (OLR1) in HCC cells. Furthermore, *in vivo* study demonstrated that rutin could inhibit autophagy through the BANCR/miRNA-590-5P/OLR1 axis. Our findings suggest that rutin could regulate autophagy by regulating BANCR/miRNA-590-5P/OLR1 axis.

## Introduction

Hepatocellular carcinoma (HCC) is one of the most common malignancies all over the world 1, 2. It is worth noting that chemotherapy resistance reduces the sensitivity of HCC to chemotherapy drugs and limits the clinical application of chemotherapy 3,4. The current effectiveness of various chemotherapy regimens is only about 30%, and multi-drug resistance is a common problem 5. Sorafenib (SO) is the cornerstone drug for the treatment of HCC patients and can significantly inhibit cancer progression 6. However, the key mechanism by which autophagy mediates SO-resistance in HCC remains unclear.

Autophagy is a physiological process that is ubiquitous in normal cells and HCC cells 7-9. When the cells become cancerous, the level of autophagy increases under the stimulation of various pathological factors such as hypoxia, starvation, radiotherapy, and chemotherapy 9. Its protective effect makes the cells better adapt to the harsh living environment to maintain survival, promote tumor progression and produce multi-drug resistance. After chemotherapy drugs act on cancer cells, they induce protective autophagy and inhibit apoptosis 9. Several studies have pointed out that a certain level of autophagy persists in the HCC cell line HepG2, and low-dose SO can up-regulate the expression of microtubule-associated protein 1 light chain 3 (LC3)-II 10, 11. Under the electron microscope, it can also be found that SO causes increased autophagy vesicles in HCC cells, all of which can prove that SO causes enhanced autophagy in HCC cells 11.

Studies have pointed out that the abnormal regulation of long non-coding RNA (lncRNA) can promote the occurrence and development of tumors, and can also promote the resistance of tumor cells to anti-tumor drugs by promoting or inhibiting autophagy 12-15. Previous studies have reported that overexpression of lncRNA BRAF-activated non-protein coding RNA (BANCR) acted as an unfavorable prognostic biomarker in HCC patients 16, 17. Rutin, the main component of *Potentilla discolor* Bunge, has anti-inflammatory and analgesic, antibacterial, antioxidant, hypoglycemic, antidiarrheal, immunosuppressive, and anti-tumor effects 18, 19. In traditional Chinese medicine, it has been used to cure inflammation 20, edema 21, mastitis 22, and gastric cancer 23. However, the applications of rutin in HCC treatment and chemotherapy resistance are rarely reported.

In this study, we showed that rutin reversed SO resistance by inhibiting autophagy through the BANCR/miRNA-590-5P/oxidized low-density lipoprotein receptor 1 (OLR1) axis in two HCC cell lines. We generated SO-resistant HCC cell lines HepG2/SO and HCCLM3/SO, and confirmed that rutin can inhibit autophagy by inhibiting the expression of BANCR and regulating the BANCR/miRNA-590-5P/OLR1 axis. *In vivo* experiments further showed that rutin exerted marked anti-tumor effects, indicating that this drug has great potential for the treatment of HCC patients with SO resistance.

## Material and methods

### Cell lines, cell culture, and transfection

Human HCC cell lines [Hep3B (RRID: CVCL_XE51), HepG2 (RRID: CVCL_0027), Huh-7 (RRID: CVCL_0336), HCCLM3 (RRID: CVCL_6832), and SK-HEP-1 (RRID: CVCL_0525)] were purchased from the Chinese Academy of Medical Sciences (Beijing, China). Hep3B, HepG2, Huh-7, HCCLM3, and SK-HEP1 cells were cultured in RPMI 1640 Medium (Invitrogen, Carlsbad, CA, USA) supplemented with 10% FBS (Invitrogen, Carlsbad, CA, USA) and 1% penicillin/streptomycin at 37 °C in 5% CO_2_.

Small interference RNAs (si-RNAs) targeting miRNA-590-5P (si-miRNA-590-5P) and OLR1 (si-OLR1), scramble control (si-control), miRNA-590-5P mimic, and its scramble control (miR-control) were obtained from GenePharma Co. Ltd (Suzhou, China). All these plasmids were transfected into HepG2 or HCCLM3 cells by Lipofectamine^®^ 2000 reagent (Invitrogen, Carlsbad, CA, USA).

### Reagents, Drugs, and antibodies

Rutin (purity ≥99%) product was purchased from National Institutes for Food and Drug Control (Beijing, China). DMSO was purchased from Sigma (St. Louis, MO, USA). SO was purchased from Sigma-Aldrich Co. ltd. (St. Louis, MO, USA). For* in vivo* experiments, SO and rutin were diluted with sterile saline. 3-Methyladenine (3-MA) was purchased from MedChem Express (Shanghai, China). The lentiviral vectors expressing human lncRNA BANCR gene (Lv-BANCR) or shRNA targeting BANCR (sh-BANCR) were purchased from BGI (Shenzhen, China). LC3 (Masahiro Shibata Lab Osaka University Japan Cat# Shibata_LC3, RRID: AB_2716621), Beclin-1 (US Biological Cat# B0981-23T, RRID: AB_2064477), p62 (Enzo Life Sciences Cat# BML-PW1230, RRID: AB_10997589), OLR1 (US Biological Cat# L2623-01C, RRID: AB_2299036), and β-actin (Bioworld Technology Cat# AP0731, RRID: AB_2797410) primary antibodies and anti-rabbit IgG HRP-linked secondary antibody were also obtained.

### HPLC analysis

Weigh 100 mg of rutin and makeup to 100 mL with methanol. The sample injection volume is 2.0 μl, and the chromatogram is recorded at 210 nm. HPLC was carried out on the Agilent 1260 chromatograph (Agilent, USA). The separation was performed at a flow rate of 0.2 mL/min.

### Establishment of SO-resistant cell lines

SO-resistant cell lines (HepG2/SO and HCCLM3/SO) were established by exposing HepG2 and HCCLM3 cells to gradually increasing the dose of SO (0.1-2.0 μg/mL) for 5 months, and the cell viability was tested.

### Compliance with Ethical Standards

Informed consent was obtained from each patient included in the study, and the study conforms according to The Code of Ethics of the World Medical Association (Declaration of Helsinki), printed in the British Medical Journal (18 July 1964). This study was approved by the Clinical Medical Research Ethics Committee of the Taihe Hospital (Shiyan, P. R. China; Approval number: 2015-019). All animal experiments complied with protocols approved by the Ethics Committee of Taihe Hospital (Shiyan, P. R. China).

### Clinical samples and chemoresistance evaluation

Sixty-eight paired HCC and adjacent tissues were obtained from HCC patients who received the same chemotherapy treatment at Taihe Hospital. According to the HUVOS grading 24, HCC patients were classified as chemoresistance or chemosensitive responders.

### Immunohistochemical analysis

Primary antibody OLR1 (US Biological Cat# L2623-01C, RRID: AB_2299036) was added to the HCC and adjacent tissues, and the biotinylated goat anti-rabbit secondary antibody was incubated at 4 °C. Finally, the sections were incubated with DAB substrate for 5 min.

### Expression profile analysis of lncRNAs

The expression profile of lncRNAs was tested by high-throughput sequencing technology. Briefly, total RNA was extracted, and then ribosomal RNA and linear RNA were removed from the total RNA sample. The edgeR program (https://www.bioconductor.org/) was used to identify differentially expressed genes. Genes with altered expression were considered differentially expressed (*P*<0.05, and more than 2 fold-change).

### LC3-dual-fluorescence assay and transmission electron microscopy

To detect changes in the endogenous LC3, cells infected with RFP-GFP-LC3 double fluorescence lentivirus (Hanbio, Shanghai, China) were cultured on coverslips in 24-well plates. Fluorescence images were directly taken using an inverted confocal microscope (Olympus, Japan). Autophagic flux was examined by confocal counting of the GFP+/RFP+ or GFP-/RFP+ puncta in cells (Excitation wavelength: 488 nm; Emission wavelength: 509 nm).

Cells were fixed with 2.5% glutaraldehyde in phosphate buffer. The specimens were post-fixed in 1% Osmium Tetroxide with 0.1% potassium ferricyanide, dehydrated through a graded series of ethanol (30%-90%). Images were taken on a HITACHI HT7700 transmission electron microscopy system (HITACHI, Japan) at 80 kV.

### 5-ethynyl-20-deoxyuridine assay

HepG2/SO and HCCLM3/SO cells were incubated with 5-ethynyl-20-deoxyuridine (EdU, Shanghai Huicheng Biological Technology Co., Ltd., Shanghai, China) for 5 hours. After three washes with phosphate buffer saline, cells were treated with a 1 × Apollo® reaction cocktail (100 μl/well) for 30 min. After that, each well was stained with Hoechst 33342 (5 μg/ml, 100 µl/well) for 30 min.

### Cell apoptosis assay

Cell apoptosis was determined by flow cytometry after staining with Annexin V (FITC-conjugated) apoptosis kit (Cat: P-CA-103, Wuhan Promise Life Technology Co., Ltd.). Briefly, HCC cells were stained with 500 µl binding buffer and 5 µl Annexin 5-FITC and propidium iodide, respectively, and incubated for 15 min in the dark, and then analyzed by flow cytometry.

### Luciferase reporter assay

BANCR and OLR1 3'UTR containing miRNA-590-5P binding sites were constructed into pGL3 vectors (Shanghai Yihui Biological Technology Co., Ltd., Shanghai, China) to form a wide type (WT)-BANCR and OLR1 3'UTR-WT. Mutant type (Mu)-BANCR and OLR1 3'UTR-Mu were generated through GeneArt™ Site-Directed Mutagenesis System (Invitrogen, Carlsbad, CA, USA). Then, they were co-transfected with pRL-TK vectors (Shanghai Yihui Biological Technology Co., Ltd., Shanghai, China) and miRNA-590-5P mimic or miR-control into HepG2 and HCCLM3 cells.

### Cell viability assay

HCC cells were seeded into 96-well plates. Culture medium was replaced with media containing different concentrations of SO (0, 0.5, 0.75, 1, and 2 μg/ml) or 3-MA (5 mM) for 48 h. Cell viability was assessed using a cell counting kit-8 assay (Shanghai Yihui Biological Technology Co., Ltd., Shanghai, China). The equation of half-maximal inhibitory concentration (IC50) of SO was [viability% = 100/(1 + 10^[SO concentration]log IC50^)].

### Quantitative real-time polymerase chain reaction (qRT-PCR)

Total RNA of cell lines was by TRIzol (15576425, Invitrogen, USA), and then cDNA was further synthesized by PrimeScriptTM RT reagent Kit (RR039A, TaKaRa, Japan). Pre-synthesized gene primers (Sangon, China), Roche SYBR Green Master (05629017212), and DEPC water were added to the cDNA and mixed together and tested in the detection instrument (thermal cycler T100, Bio-Rad, USA), according to the following settings: pre-denaturation at 95 °C for 10 minutes (min), denaturation at 95 °C for 15 s, annealing at 58 °C for 1min, for a total of 40 cycles. For calculating RNA levels, glyceraldehyde-3-phosphate dehydrogenase (GAPDH) and U6 were internal references. The primer's sequences were shown in Table 1. The amplification efficiencies of BANCR, miRNA-590-5P, LC3-I, LC3-II, Beclin-1, P62, OLR1, GAPDH, and U6 were 97.2%, 94.6%, 103.7%, 101.3%, 95.0%, 99.5%, 104.7%, 96.0%, and 98.1%, respectively. The relative level was calculated by the 2^-△Ct^ method.

### Western blot

The primary and secondary antibodies used in the experiment were purchased from Abcam. The proteins of LC3, Beclin-1, P62, OLR1, and β-actin were tested by Western blot. The rabbit anti-LC3 (1:2000, ab128025, Abcam, USA), Beclin-1 (1:2000, ab39729, Abcam, USA), P62 (1:1000, ab91526, Abcam, USA), OLR1 (1:1000, ab119603, Abcam, USA), and β-actin (1:3000, ab8227, Abcam, USA) antibodies were added overnight at 4 °C. The secondary antibody was then added and incubated at room temperature for 0.5 h the next day.

### RNA pull-down assay

The RNA sequences used for RNA pull-down assay were transcribed from their corresponding plasmids *in vitro*, and biotin-labeled using the Biotin RNA Labeling Mix (Roche, 11685597910). Total RNAs from HepG2 cell extracts were mixed with biotin-labeled miRNA-590-5P, incubated with Dynabeads M-280 Streptavidin (Invitrogen, 11205D) at 4 °C for 3h. Finally, the expression of BANCR and OLR1 contained in the pulled-down complexes were tested by qRT-PCR.

### *In vivo* tumor xenograft

Male BALB/c nude mice (12-14 g, 3-4 weeks old) were obtained from Silaike Experimental Animal Centre (Shanghai, China). Humane care was given during the experimental animal breeding and experimental procedures following the 3R principle of experimental animals. Twenty-four mice were subcutaneously injected (right axillary fossa) with 1×10^6^ HCCLM3/SO cells in 100μl phosphate buffer saline. The tumor volume was calculated regularly according to the formula: Volume of tumor (mm^3^) = (length × width^2^)/2. Tumor volumes were calculated every other day. Mice received SO (3 mg/kg), rutin (3 mg/kg), SO (3 mg/kg) combined with rutin (3 mg/kg), or equal volume of phosphate buffer saline (control group) intraperitoneally every 2 days. After 14 days, mice were euthanized by cervical dislocation.

### Statistical analyses

All experiments were performed in triplicate unless specified. Results were represented as the Mean ± SEM. The differences between normally distributed numeric variables were evaluated by Student's t-test, whereas non-normally distributed variables were analyzed by Mann-Whitney U-test. One-way ANOVA was used for the comparison among multiple groups if the variance was homogeneous, while non-normally distributed variables were evaluated by Kruskal-Wallis variance analysis. Multiple comparisons between the groups were performed using the S-N-K method. Correlations were analyzed using the Pearson correlation analysis. *P*<0.05 was considered significant.

## Results

### Characterization of SO resistance in HepG2/SO and HCCLM3/SO cells

**Figure 1:**
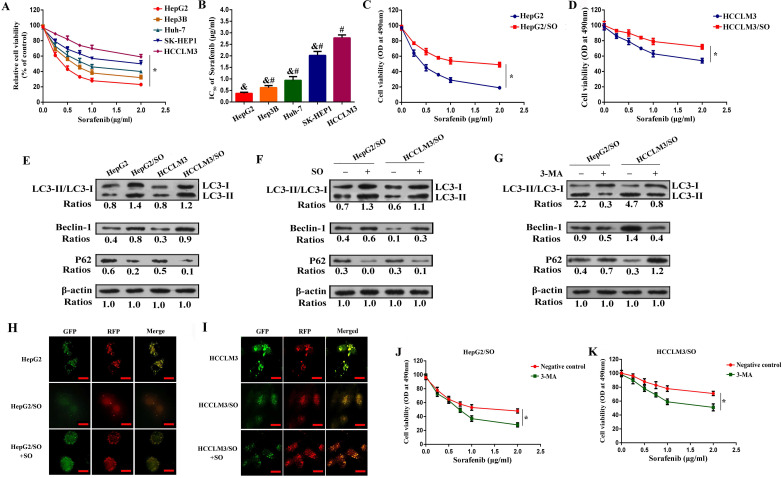
Autophagy level was increased in HepG2/SO and HCCLM3/SO cells. **(A-B)** SO cytotoxicity for Hep3B, HepG2, Huh-7, HCCLM3, and SK-HEP-1 cell lines was examined by cell viability assay. * One-way ANOVA was used, and *P*<0.05; # Compared with HepG2 group, *P*<0.05; & Compared with HepG2 group, *P*<0.05. **(C)** The IC50 for HepG2/SO cells was examined by cell viability assay. *One-way ANOVA was used, and *P*<0.05. **(D)** The IC50 for HCCLM3/SO cells was examined by cell viability assay. * One-way ANOVA was used, and *P*<0.05. **(E)** The Beclin-1 level and LC3-II/LC3-I ratio dramatically increased, while the P62 level prominently decreased in HepG2/SO and HCCLM3/SO cells, as compared with the parental cells. **(F)** Treatment with SO (1.93±0.27 µg/ml for HepG2/SO cells; 5.06±0.89 µg/ml for HCCLM3/SO cells) for 24h resulted in more significant changes in the autophagy biomarkers. **(G)** Treatment with 5mM 3-MA for 24 h prominently restored SO sensitivity in HepG2/SO and HCCLM3/SO cells, verified by the decrease in IC50 value. **(H-I)** The RFP-GFP-LC3 in autolysosomes was increased in SO-treated HCCLM3/SO and HepG2/SO cells tested by fluorescence microscope. * Compared with RFP+GFP+ group, *P*<0.05. **(J-K)** Treatment with 5mM 3-MA for 24 h prominently restored SO sensitivity in HepG2/SO and HCCLM3/SO cells (cell viability assay was used), verified by the decrease in IC50 value. * One-way ANOVA was used, and *P*<0.05. SO: Sorafenib; IC50: half maximal inhibitory concentration; 3-MA: 3-Methyladenine. The scale bar is 10 um.

SO cytotoxicity for Hep3B, HepG2, Huh-7, HCCLM3, and SK-HEP-1 cell lines was examined. HepG2 cells possessed the most sensitivity to SO accompanied by the lowest IC50, whereas HCCLM3 cells were found to be the least sensitive accompanied by the highest IC50 (Figure 1A-B). Furthermore, we characterized SO resistance in HepG2/SO and HCCLM3/SO cells. After exposure to gradually increasing dose of SO (0.1-2.0 μg/mL) for 2 days, the IC50 values were 1.93±0.27 μg/ml and 5.06±0.89 μg/ml for HepG2/SO and HCCLM3/SO cells, respectively, showing a 4.7- and 1.9-fold increase as compared to parental cell lines (HepG2 cells: 0.41±0.11 μg/ml, Figure 1C; HCCLM3 cells: 2.72±0.50 μg/ml, Figure 1D).

It has been documented that higher Beclin-1 expression and LC3-II/LC3-I ratio, and lower P62 level indicated stronger autophagy 25. Our results indicated that the Beclin-1 level and LC3-II/LC3-I ratio dramatically increased, while the P62 level prominently decreased in HepG2/SO and HCCLM3/SO cells (Figure 1E), as compared with the parental cells, indicating that autophagic activity is enhanced in HepG2/SO and HCCLM3/SO cells. Moreover, treatment with SO (1.93±0.27 μg/ml for HepG2/SO cells; 5.06±0.89 μg/ml for HCCLM3/SO cells) for 24h resulted in more significant changes in the autophagy biomarkers, thereby indicating a further increased autophagy level in response to SO in HepG2/SO and HCCLM3/SO cells (Figure 1F). Interestingly, a fluorescence microscope further confirmed our findings, evidenced by increased RFP-GFP-LC3 in autolysosomes in SO-treated HCCLM3/SO and HepG2/SO cells (Figure 1H-I). We also found that treatment with 5mM 3-MA (an autophagy inhibitor) for 24 h prominently restored SO sensitivity in HepG2/SO and HCCLM3/SO cells, verified by the decrease in IC50 value (HepG2/SO: from 1.93±0.27 μg/ml to 0.76±0.13 μg/ml; HCCLM3/SO: from 5.06±0.89 μg/ml to 1.73±0.18 μg/ml, Figure 1G, J, K).

### Expression profile analysis of lncRNAs between SO-sensitive and -resistant HCC cells

**Figure 2:**
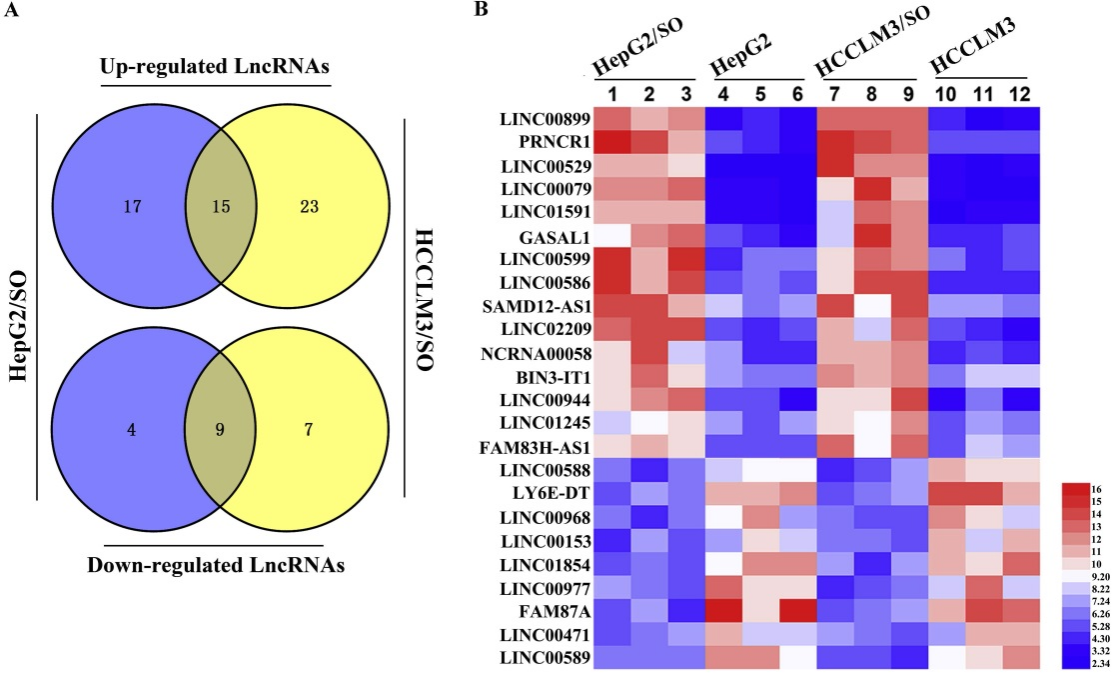
Expression profile of lncRNAs was tested between SO-sensitive and -resistant HCC cells. **(A)** The Venn diagram revealed an intersection of differentially expressed lncRNAs between the two cell groups. **(B)** Heat map of the intersection of 29 differentially expressed lncRNAs between the two cell groups, of which LINC00586 refers to BANCR. The red color represents a high fold change while the blue color represents a low fold change. SO: Sorafenib; lncRNA: long non-coding RNA; BANCR: BRAF-activated non-protein coding RNA.

Next, the expression profile of lncRNAs between SO-sensitive and -resistant HCC cells was analyzed through high-throughput RNA-seq technology. Briefly, 45 lncRNAs were differentially expressed between HepG2 and HepG2/SO cells, of which 13 were down-regulated and 32 were up-regulated. Between HCCLM3 and HCCLM3/SO cells, 54 lncRNAs were differentially expressed, of which 16 were down-regulated and 38 were up-regulated. The Venn diagram and heat map were shown in Figure 2A-B.

### Expression of BANCR in SO-sensitive and -resistant cells and tissue samples, and its correlation with autophagy markers

**Figure 3:**
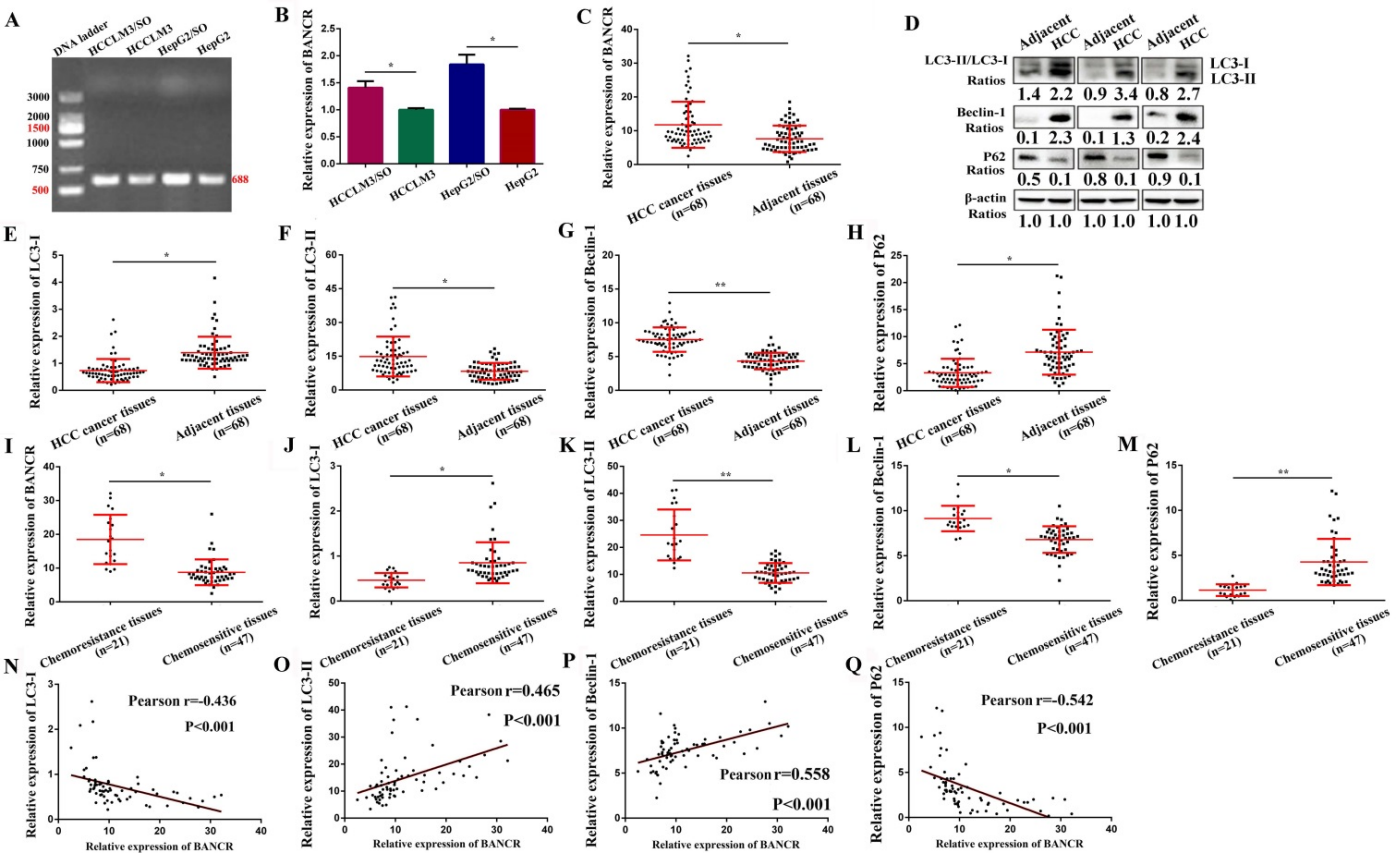
BANCR level was increased in SO-resistant cells and tissues, and was correlated with autophagy markers. **(A-B)** BANCR expression was significantly up-regulated in HepG2/SO and HCCLM3/SO cells, compared to parental cell lines. * Compared with HepG2/HCCLM3 group, *P*<0.05. **(C-H)** BANCR, Beclin-1 levels, and LC3-II/LC3-I ratio were up-regulated in HCC tissues, while the P62 level was down-regulated, compared to adjacent tissues (qRT-PCR and Western blot were used). * Compared with adjacent tissues, *P*<0.05. ** Compared with adjacent tissues, *P*<0.01. **(I-M)** BANCR, Beclin-1 levels, and LC3-II/LC3-I ratio were up-regulated, while the P62 level was down-regulated in chemoresistance tissues than in chemosensitive tissues (qRT-PCR was used). * Compared with chemosensitive tissues, *P*<0.05. ** Compared with chemosensitive tissues, *P*<0.01. **(N-Q)** BANCR expression was correlated with autophagy markers (Pearson correlation analysis was used). SO: Sorafenib; lncRNA: long non-coding RNA; BANCR: BRAF-activated non-protein coding RNA. Data were expressed as mean ± SEM.

Among the 15 up-regulated lncRNAs, LINC00586, also refers to BANCR, is associated with apoptosis and the cell cycle of HCC cells 16, 17. Therefore, we speculate that up-regulation of BANCR may be involved in the development of SO resistance in HCC. We found that BANCR expression was significantly up-regulated in HepG2/SO and HCCLM3/SO cells, compared to parental cell lines (Figure 3A-B). Moreover, BANCR, Beclin-1 levels, and LC3-II/LC3-I ratio were up-regulated in HCC tissues, while the P62 level was down-regulated, compared to adjacent tissues (Figure 3C-H). Similarly, BANCR, Beclin-1 levels, and LC3-II/LC3-I ratio were up-regulated, while the P62 level was down-regulated in chemoresistance tissues than in chemosensitive tissues (Figure 3I-M). Further analysis indicated that BANCR was positively correlated with autophagic activity (Figure 3N-Q).

### Rutin treatment attenuates autophagy and BANCR expression in HepG2/SO and HCCLM3/SO cells

**Figure 4:**
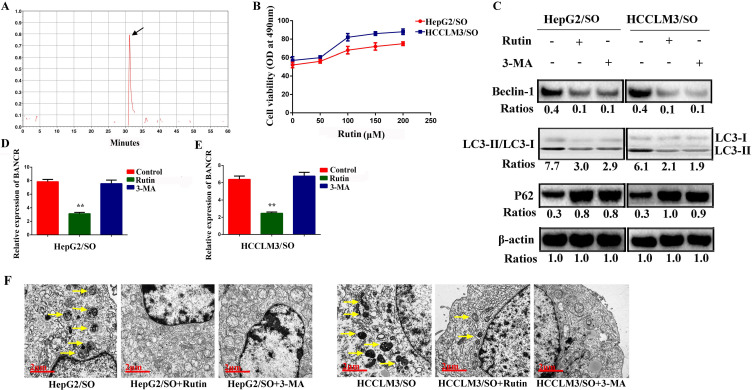
Rutin treatment attenuates autophagy and BANCR expression in HepG2/SO and HCCLM3/SO cells. **(A)** HPLC analysis results showed that the retention time for rutin was around 30 min. **(B)** Rutin significantly inhibited HepG2/SO and HCCLM3/SO cell growth in a dose-dependent manner (cell viability assay was used). **(C-E)** Treatment with 5mM 3-MA or 75 µM rutin for 24h both prominently restored SO sensitivity in HepG2/SO and HCCLM3/SO cells, verified by the decrease in BANCR, Beclin-1 levels and LC3-II/LC3-I ratio, and increase in P62 (qRT-PCR and Western blot were used). One-way ANOVA was used, ** Compared with the control or 3-MA group, *P*<0.05. **(F)** Morphology observation by transmission electron microscopy demonstrated a significantly decreased number of autophagosomes after 3-MA or rutin-treated HepG2/SO and HCCLM3/SO cells. SO: Sorafenib; BANCR: BRAF-activated non-protein coding RNA. The scale bar is 2 um.

The retention time for rutin was around 30 min (Figure 4A). HepG2/SO and HCCLM3/SO cells were treated with rutin. The results showed that the rutin significantly inhibited HepG2/SO and HCCLM3/SO cell growth in a dose-dependent manner (Figure 4B). Based on the above results, we selected rutin at 75 μM as the optimal concentration. We also found that treatment with 5mM 3-MA or 75 μM rutin for 24h both prominently restored SO sensitivity in HepG2/SO and HCCLM3/SO cells, verified by the decrease in BANCR, Beclin-1 levels and LC3-II/LC3-I ratio, and increase in P62 (Figure 4C-E). Furthermore, morphology observation by transmission electron microscopy clearly demonstrated a significantly decreased number of autophagosomes after 3-MA or rutin-treated HepG2/SO and HCCLM3/SO cells (Figure 4F).

### BANCR knockdown promotes the sensitivity of HepG2/SO and HCCLM3/SO cells to SO

**Figure 5:**
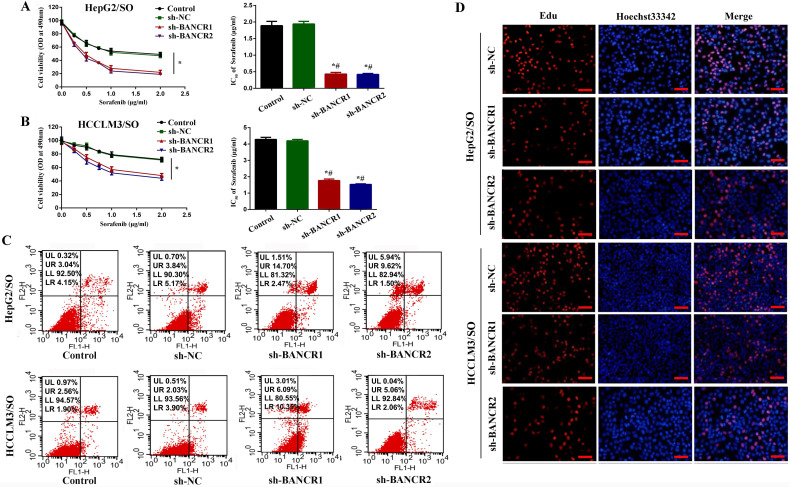
BANCR knockdown promotes the sensitivity of HepG2/SO and HCCLM3/SO cells to SO. **(A)** Under SO treatment, the IC50 value of the sh-BANCR group (HepG2/SO) was significantly down-regulated, compared to the control group and the sh-NC group (cell viability assay was used). One-way ANOVA was used. * Compared with the control group or sh-NC group, *P*<0.05; # Compared with the control group or sh-NC group, *P*<0.05. **(B)** Under SO treatment, the IC50 value of the sh-BANCR group (HCCLM3/SO) was significantly down-regulated, compared to the control group and the sh-NC group (cell viability assay was used). One-way ANOVA was used. * Compared with the control group or the sh-NC group, *P*<0.05; # Compared with the control group or sh-NC group, *P*<0.05. **(C)** BANCR knockdown enhanced cell apoptosis in HepG2/SO and HCCLM3/SO cells. **(D)** BANCR knockdown inhibited the number of EdU-positive cells in HepG2/SO and HCCLM3/SO cells. SO: Sorafenib; BANCR: BRAF-activated non-protein coding RNA. The scale bar is 50 um.

To investigate the specific function of BANCR on SO-induced autophagy in HepG2/SO and HCCLM3/SO cells, we achieved BANCR silencing by infecting cells with sh-BANCR. As shown in Figure 5A-B, under SO treatment, the IC50 value of the sh-BANCR group was significantly down-regulated (0.37 μg/ml for HepG2/SO and 1.83 μg/ml for HCCLM3/SO), compared to the control group and sh-NC group. Then we further studied the effects of BANCR on HCC cell growth *in vitro*. We found that BANCR knockdown enhanced cell apoptosis (Figure 5C) and inhibited the number of EdU-positive cells (Figure 5D) in HepG2/SO and HCCLM3/SO cells.

### LncRNA BANCR acts as a molecular sponge of miRNA-590-5P to sequester miRNA-590-5P away from OLR1 in HCC cells

**Figure 6:**
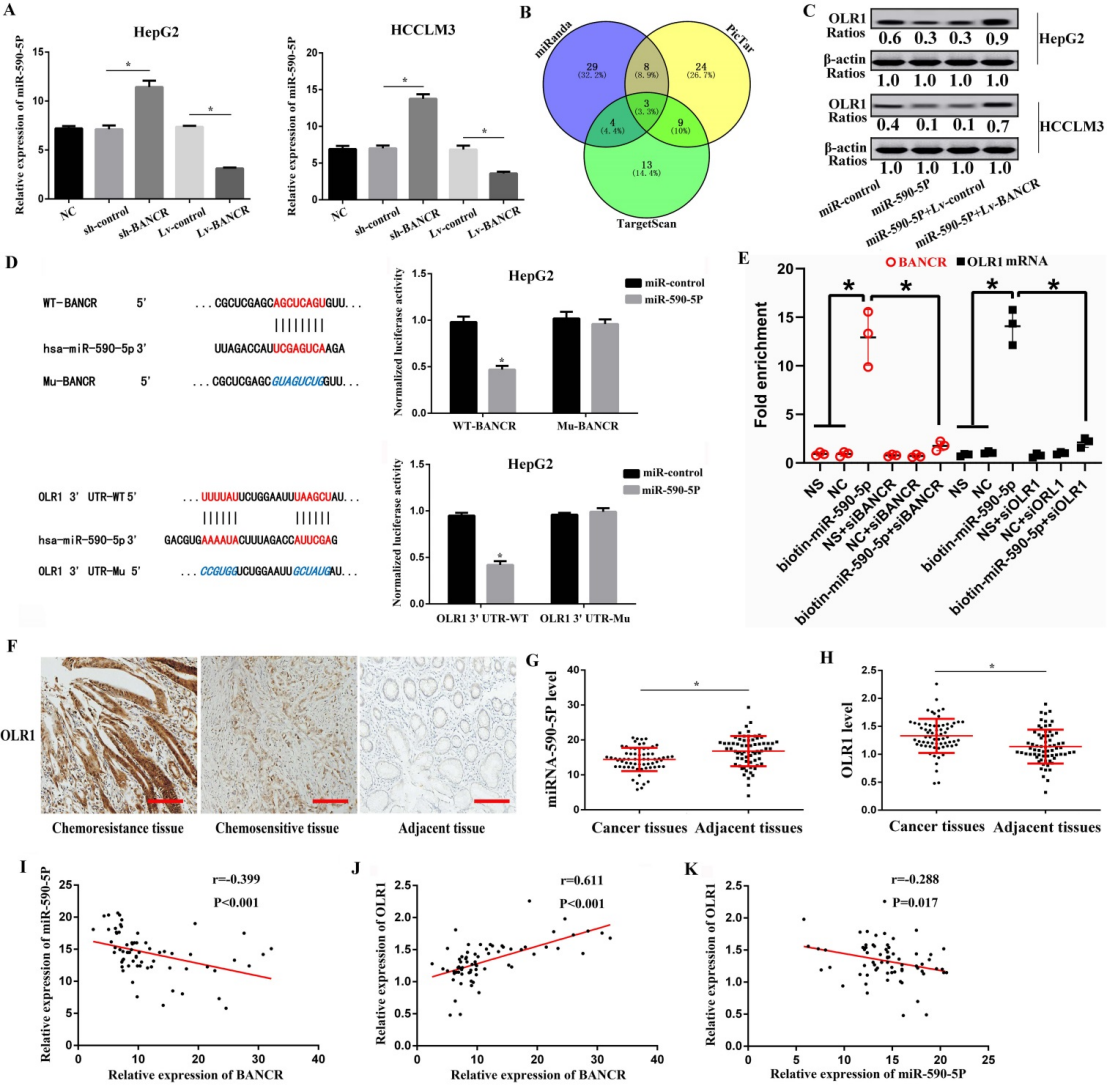
LncRNA BANCR acts as a molecular sponge of miRNA-590-5P to sequester miRNA-590-5P away from OLR1 in HCC cells. **(A)** The miRNA-590-5P level was markedly increased in the sh-BANCR group, but was prominently decreased in the Lv-BANCR group. * Compared with the control group, *P*<0.05. **(B)** Venn diagram was used to analyze the target proteins through PicTar, miRanda, and Targetscan online prediction systems. A total of 3 target protein was predicted, namely OLR1, GATA3, and Gab2. **(C)** Western blot results confirmed that miRNA-590-5P up-regulation suppressed OLR1 level, while this effect was reversed by BANCR up-regulation in HepG2 and HCCLM3 cells. **(D)** The effects of miRNA-590-5P overexpression on luciferase activity of WT-BANCR and Mu-BANCR reporters or OLR1 3'UTR‐WT and OLR1 3'UTR‐Mu reporters were determined in HepG2 cell. * Compared with the miR-control group, *P*<0.05. **(E)** RNA pull-down assay was used, and we found that BANCR and OLR1 could specifically interact with miR-590-5p (qRT-PCR was used). NS was the biotin-labeled non-sense RNA with a similar length to miR-590-5p, NC was the miR-590-5p without biotin label. One-way ANOVA was used. * Compared with the biotin-miR-590-5p group, *P*<0.05. **(F)** Compared with chemosensitive tissues and adjacent tissues, the levels of OLR1 protein in chemoresistance tissues were significantly higher. One-way ANOVA was used. **(G-H)** miRNA-590-5P level dramatically decreased, while the OLR1 level prominently increased in HCC tissues (qRT-PCR was used). * Compared with adjacent tissues, *P*<0.05. **(I-K)** BANCR was positively correlated with OLR1, and negatively correlated with miRNA-590-5P in HCC tissues (Pearson correlation analysis was used). SO: Sorafenib; lncRNA: long non-coding RNA; BANCR: BRAF-activated non-protein coding RNA; OLR1: OLR1: oxidized low-density lipoprotein receptor 1. The scale bar is 100 um. Data were expressed as mean ± SEM.

Numerous pieces of evidence show that lncRNA can regulate the effect of miRNA on downstream target mRNA through sponge adsorption 26. To further explore molecular mechanisms of BANCR in HCC progression, we used the prediction website (https://genie.weizmann.ac.il/pubs/mir07/index.html) and predicted 4 potential miRNAs (miRNA-590-5P, miRNA-612, miR-195-5p, miR-204). We found that miRNA-590-5P level was markedly increased in the sh-BANCR group, but was prominently decreased in the Lv-BANCR group (Figure 6A). However, the relative expression of miRNA-612, miR-195-5p, and miR-204 in each group showed no statistical difference (*P*>0.05). Next, we respectively applied PicTar (https://pictar.mdc-berlin.de/), miRanda (http://www.microrna.org/), and Targetscan (http://www.targetscan.org/) online prediction systems, and found out the target proteins, then used Venn diagram to analyze the target proteins jointly predicted by the three software. A total of 3 target protein was predicted, namely OLR1, GATA3, and Gab2 (Figure 6B). Western blot results confirmed that miRNA-590-5P up-regulation suppressed OLR1 level, while this effect was reversed by BANCR up-regulation in HepG2 and HCCLM3 cells (Figure 6C), indicating that BANCR acted as a miRNA-590-5P sponge to sequester miRNA-590-5P away from OLR1 in HCC cells. Luciferase assays found that miRNA-590-5P overexpression prominently inhibited the luciferase activity of WT-BANCR, but had no effect on Mu-BANCR in HepG2 and HCCLM3 cells (Figure 6D). To verify the interaction between BANCR/miR-590-5p or miR-590-5p/OLR1, we performed an RNA pull-down assay. As shown in Figure 6E, the BANCR and OLR1 could specifically interact with miR-590-5p. Then, OLR1 3'UTR-WT and OLR1 3'UTR-Mu reporters were built. Luciferase assays found that luciferase activity of OLR1 3'UTR-WT was significantly inhibited in miRNA-590-5P-overexpressed HepG2 and HCCLM3 cells, but had no effect on OLR1 3'UTR-Mu reporter (Figure 6D). Immunohistochemistry results showed that compared with chemosensitive tissues and adjacent tissues, the levels of OLR1 protein in chemoresistance tissues were significantly higher (Figure 6F). QRT-PCR results indicated that miRNA-590-5P level dramatically decreased, while the OLR1 level prominently increased in HCC tissues (Figure 6G-H), as compared with the adjacent tissues. Further analysis indicated that BANCR was positively correlated with OLR1, and negatively correlated with miRNA-590-5P in HCC tissues (Figure 6I-K).

### Rutin regulates autophagy through the BANCR/miRNA-590-5P/OLR1 axis

**Figure 7:**
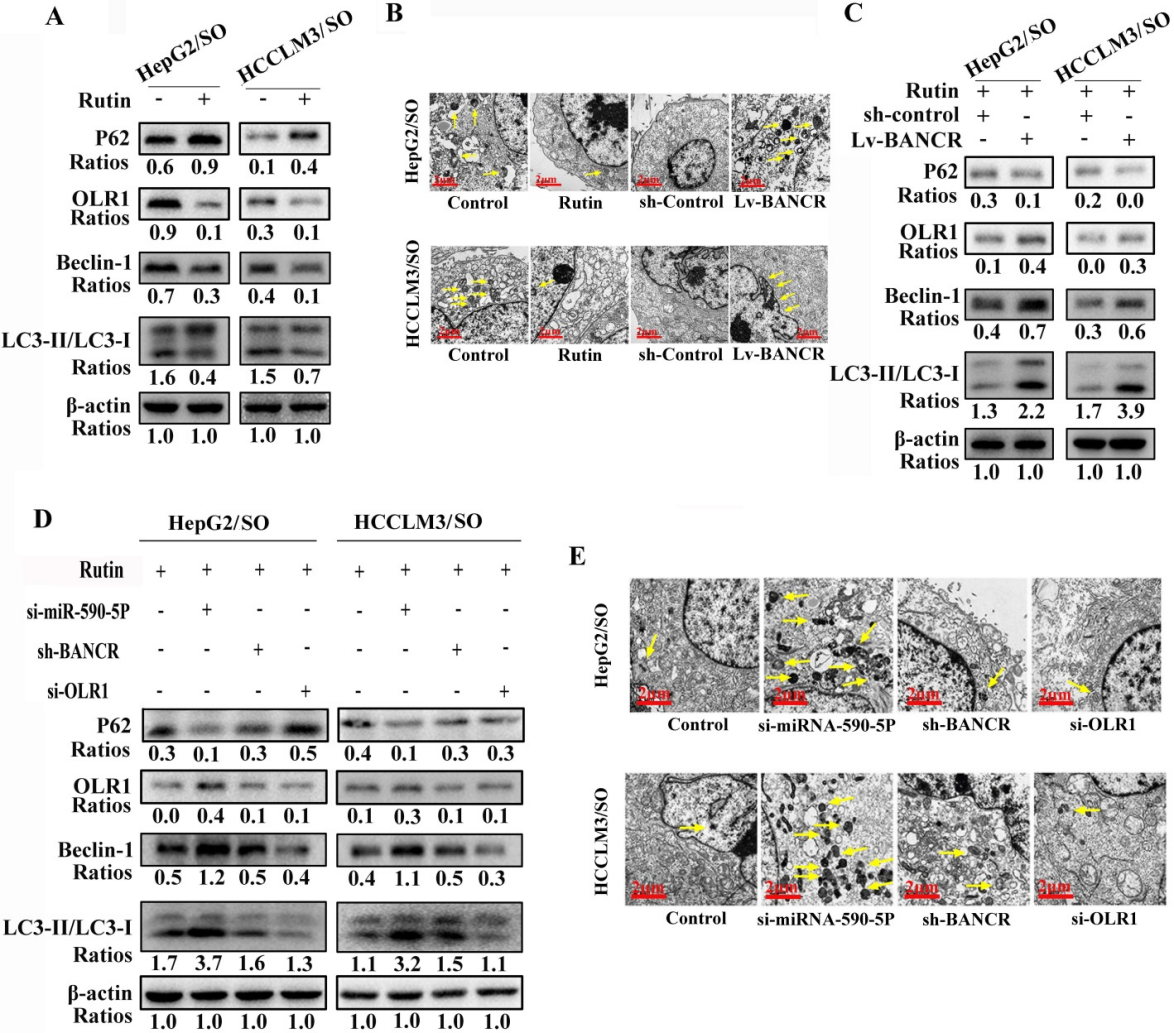
Rutin can regulate autophagy through the BANCR/miRNA-590-5P/OLR1 axis. **(A-C)** Rutin treatment inhibited the autophagic activity and OLR1 levels in HepG2/SO and HCCLM3/SO cells. Moreover, up-regulation of BANCR increased the autophagic activity in HepG2/SO and HCCLM3/SO cells. **(D-E)** After sh-BANCR or si-OLR1 treatment, the autophagic activity was weakened in HepG2/SO and HCCLM3/SO cells. The yellow arrow points to the autophagosome. SO: Sorafenib; BANCR: BRAF-activated non-protein coding RNA; OLR1: OLR1: oxidized low-density lipoprotein receptor 1. The scale bar is 2 um.

Rutin treatment inhibited the autophagic activity and OLR1 levels in HepG2/SO and HCCLM3/SO cells (Figure 7A-B). Moreover, up-regulation of BANCR increased the autophagic activity in HepG2/SO and HCCLM3/SO cells (Figure 7B-C). After si-miRNA-590-5P treatment, the autophagic activity was enhanced both in HepG2/SO and HCCLM3/SO cells (Figure 7D-E). While after sh-BANCR or si-OLR1 treatment, the autophagic activity was weakened in HepG2/SO and HCCLM3/SO cells (Figure 7D-E).

### Rutin enhances the efficacy of SO in a xenograft model of HCC in nude mice

**Figure 8:**
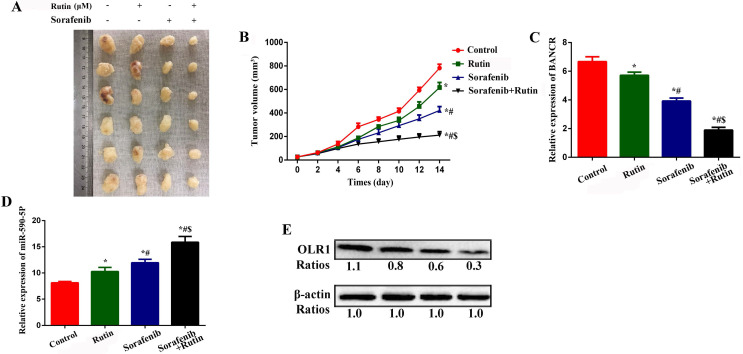
Rutin can enhance the efficacy of SO in a xenograft model of HCC in nude mice. **(A-B)** Intraperitoneal injection of rutin and SO alone for 14 days inhibited tumor growth. One-way ANOVA was used. * Compared with the control group, *P*<0.05; # Compared with the Rutin group, *P*<0.05; $ Compared with the Sorafenib group, *P*<0.05. (C) Intraperitoneal injection of rutin and SO alone for 14 days inhibited BANCR expression (qRT-PCR was used). One-way ANOVA was used. * Compared with the control group, *P*<0.05; # Compared with the Rutin group, *P*<0.05; $ Compared with the Sorafenib group, *P*<0.05. **(D)** Intraperitoneal injection of rutin and SO alone for 14 days promoted miRNA-590-5P expression (qRT-PCR was used). One-way ANOVA was used. * Compared with the control group, *P*<0.05; # Compared with the Rutin group, *P*<0.05; $ Compared with the Sorafenib group, *P*<0.05. **(E)** Intraperitoneal injection of rutin and SO alone for 14 days inhibited OLR1 expression. SO: Sorafenib; BANCR: BRAF-activated non-protein coding RNA; OLR1: OLR1: oxidized low-density lipoprotein receptor 1.

We subcutaneously injected HCCLM3/SO cells into nude mice to establish a xenograft model of HCC. Intraperitoneal injection of rutin and SO alone for 14 days inhibited tumor growth (Figure 8A-B), BANCR (Figure 8C), and OLR1 (Figure 8E) levels, while promoted miRNA-590-5P expression (Figure 8D). Interestingly, combined treatment with rutin and SO led to more significant inhibition of tumor volume, BANCR, and OLR1 levels, and promotion of miRNA-590-5P (Figure 8) expression.

## Discussion

Recent studies have shown that non-coding RNA, especially lncRNA, is closely related to the occurrence of tumor chemotherapy resistance 27-31, and BANCR is associated with chemotherapy resistance in colorectal cancer 32. However, whether BANCR is involved in the resistance of HCC to SO has not been reported.

SO is widely used for the treatment of malignant tumors 33-36. However, drug resistance of cancer cells has become a major obstacle to compromise the efficiency of SO. Drug resistance of SO has been reported in various advanced tumors, including HCC 36, breast cancer 37, and gastric cancer 38, and often results in poor prognosis.

Although the underlying mechanism of drug resistance of SO remains unclear, autophagy has been implicated to play a crucial role 7-9. It is well known that autophagy plays a dual role in tumor development. It can serve as either a tumor suppressor to inhibit tumor progression or a cell survival enhancer to promote tumor growth 39, 40. On the one hand, autophagy recycles damaged cell components and provides substrates for biosynthesis and energy metabolism 39. On the other hand, excessive autophagy may lead to over digestion of cell components, and even apoptosis of cells 40. Increased autophagy also plays a dual role in drug resistance depending, to a large extent, on the type of tumor and the nature of metabolic stress induced by the specific treatment 41, 42. Numerous studies have demonstrated that increased autophagy promotes cell survival and results in SO resistance 9. For example, increased autophagy and decreased apoptosis were observed in SO-resistant multiple myeloma cells 43, and treatment with SO induced cytoprotective autophagy in breast cancer cells 37. Concerning the role of autophagy in SO-resistant HCC, documents are still limited.

In this study, we established two HCC cell lines (HepG2/SO and HCCLM3/SO) with acquired SO-resistant by using the concentration gradient exposure method. We found that the IC50 value and autophagic activity were increased in HepG2/SO and HCCLM3/SO cells compared with their parental cell lines. Our finding demonstrates that elevated autophagy emerges in response to SO in HCC cells, which might be a self-protective against apoptosis 9. In addition, we also found that 3-MA, the autophagy inhibitor, could reverse SO-resistance in HepG2/SO and HCCLM3/SO cells. Our findings suggesting that inhibition of autophagy may be an effective way for overcoming the SO-resistance in HCC. Another highlight in our study was that we identified BANCR as a key regulator of SO-resistance in HCC cells, which was highly expressed in HepG2/SO and HCCLM3/SO cell lines and may promote SO-resistance by increasing autophagic activity. Interestingly, we also found that BANCR, Beclin-1 levels, and LC3-II/LC3-I ratio were up-regulated, while the P62 level was down-regulated in chemoresistance tissues than in chemosensitive tissues, and BANCR was positively correlated with autophagic activity. These findings suggested that BANCR may contribute to the development of drug resistance. To explore the value of rutin on autophagy-induced SO resistance, HepG2/SO and HCCLM3/SO cells were treated with rutin. We also found that treatment with rutin prominently restored SO sensitivity in HepG2/SO and HCCLM3/SO cells, verified by the decrease in BANCR, Beclin-1 levels, and LC3-II/LC3-I ratio, and increase in P62. Transmission electron microscopy also clearly showed a significantly decreased number of autophagosomes after rutin-treated HepG2/SO and HCCLM3/SO cells. The above results initially suggested that rutin may inhibit autophagy-induced SO-resistance in HCC cells by down-regulating BANCR expression.

Autophagy is a complicated process regulated by more than 30 genes and multiple pathways by which cells maintain homeostasis. Recent studies revealed that autophagy in tumor cells could also be regulated by non-coding RNAs 44. For example, lncRNA LINC00160 was proved to function as a tumor suppressor by inhibiting autophagy and drug resistance in HCC through miR-132/PIK3R3 pathway 45, while lncRNA H19 can serve as a tumor enhancer by inducing autophagy activation via the H19/SAHH/DNMT3B axis, and contributes to tamoxifen resistance in breast cancer 46. Similarly, in our study, we reported that autophagy in HCC could be regulated by lncRNA BANCR through miRNA-590-5P/OLR1 pathway, by this approach, lncRNA BANCR also contributes to SO-resistance. These results suggest that BANCR can serve as the target to reduce SO-resistance in HCC.

Rutin, an ingredient extracted from the traditional Chinese medicine *Potentilla discolor Bunge*, exhibits a wide range of pharmacological effects 20-23. Rutin has been reported to have an autophagy inhibition effect in several kinds of cells. For instance, Ma et al. 47 found that rutin attenuates SO-induced autophagy in cardiomyocytes mediated by Akt activation. *In vivo* study also proved that rutin exhibits anti-autophagic effects and attenuates gentamicin-induced nephrotoxicity in rats 48. However, the function of rutin in SO-resistant HCC has not been reported yet. In the current study, for the first time, we investigated the role of rutin in SO-resistant HCC and found that rutin prominently restored SO sensitivity in HepG2/SO and HCCLM3/SO cells by targeting BANCR and inhibiting autophagy. The effects of rutin were further verified by the evidence from *in vivo* study that combined use of rutin and SO exhibited more significant inhibition of tumor growth. These results indicate that rutin is an efficient extract for the attenuation of autophagy-induced SO-resistant in HCC.

In conclusion, this study demonstrated the regulation of autophagy-induced SO-resistant in HCC by BANCR/miRNA-590-5P/OLR1 pathway and verified that rutin attenuated SO-resistant in HCC via targeting BANCR, which may provide a novel treatment for SO-resistant HCC.

## Figures and Tables

**Table 1:** The primer's sequences

	Primer	Sequence
Transcripts	BANCR forward	5'-CTGATGAACCCGAGCTAG-3'
	BANCR reverse	5'-GGAGCTACTAACACTCGG-3'
	miRNA-590-5P forward	5'-CTCAGATGCATTGCA-3'
	miRNA-590-5P reverse	5'-GTACCTGATGGCACCG-3'
	LC3-I forward	5'-CGCAGTACGCTATCTCGAA-3'
	LC3-I reverse	5'-GAGTGATGGACGTTCCT-3'
	LC3-II forward	5'-GACGAGGGCGATACGAAC-3'
	LC3-II reverse	5'-AGAGCCTCGCGTTAGC-3'
	Beclin-1 forward	5'-ATGCCGTACTGAATCGGCAC-3'
	Beclin-1 reverse	5'-ATCGGCATCCGATTCCG-3'
	P62 forward	5'-CGTACTAGGATCAG-3'
	P62 reverse	5'-CGCTCACCATTGCGATTG-3'
	OLR1 forward	5'-GTGCCGATTCGCACGGT-3'
	OLR1 reverse	5'-GATGGCTTACGCATCGCC-3'
	GAPDH forward	5'-GCGTCATGCGATAGAGCTA-3'
	GAPDH reverse	5'-GTCACTACCCTGCTAGCA-3'
	U6 forward	5'-CAGCTCGCGGAGGCACGC-3'
	U6 reverse	5'-ACTCTCGCTCGCATGATC-3'
siRNAs	siNC forward	5'-UUCUCCGAACGUGUCACGUdTdT-3'
	siNC reverse	5'-ACGUGACACGUUCGGAGAAdTdT-3'
	si-miRNA-590-5P forward	5'-CGTGCGGCACTCCGGACAGUCdTdT-3'
	si-miRNA-590-5P reverse	5'-AGCCGGUGGGACTGCAUUCGdTdT-3'
	si-OLR1 forward	5'-AUCGAGAAUGCUGAUACACAAdTdT-3'
	si-OLR1 reverse	5'-UUGUGUGUCAACAUUCUCGCUdTdT-3'

## Abbreviations

BANCR: BRAF-activated non-protein coding RNA
HCC: hepatocellular carcinoma
IC50: half-maximal inhibitory concentration
LC3: light chain 3
lncRNA: long non-coding RNA
miRNA: microRNA
OLR1: oxidized low-density lipoprotein receptor 1
qRT-PCR: quantitative real-time polymerase chain reaction
Mu: mutant type
si-RNAs: small interference RNAs
SO: sorafenib
WT: wide type
